# Evaluating Structural Variation Detection Tools for Long-Read Sequencing Datasets in *Saccharomyces cerevisiae*

**DOI:** 10.3389/fgene.2020.00159

**Published:** 2020-03-09

**Authors:** Mei-Wei Luan, Xiao-Ming Zhang, Zi-Bin Zhu, Ying Chen, Shang-Qian Xie

**Affiliations:** ^1^ Key Laboratory of Genetics and Germplasm Innovation of Tropical Special Forest Trees and Ornamental Plants (Ministry of Education), Hainan Key Laboratory for Biology of Tropical Ornamental Plant Germplasm, College of Forestry, Hainan University, Haikou, China; ^2^ College of Grassland, Resources and Environment, Inner Mongolia Agricultural University, Huhhot, China; ^3^ State Key Laboratory of Ophthalmology, Zhongshan Ophthalmic Center, Sun Yat-sen University, Guangzhou, China

**Keywords:** structural variation, long-read sequencing, PacBio and ONT, SV caller, Saccharomyces cerevisiae

## Abstract

Structural variation (SV) represents a major form of genetic variations that contribute to polymorphic variations, human diseases, and phenotypes in many organisms. Long-read sequencing has been successfully used to identify novel and complex SVs. However, comparison of SV detection tools for long-read sequencing datasets has not been reported. Therefore, we developed an analysis workflow that combined two alignment tools (NGMLR and minimap2) and five callers (Sniffles, Picky, smartie-sv, PBHoney, and NanoSV) to evaluate the SV detection in six datasets of *Saccharomyces cerevisiae*. The accuracy of SV regions was validated by re-aligning raw reads in diverse alignment tools, SV callers, experimental conditions, and sequencing platforms. The results showed that SV detection between NGMLR and minimap2 was not significant when using the same caller. The PBHoney was with the highest average accuracy (89.04%) and Picky has the lowest average accuracy (35.85%). The accuracy of NanoSV, Sniffles, and smartie-sv was 68.67%, 60.47%, and 57.67%, respectively. In addition, smartie-sv and NanoSV detected the most and least number of SVs, and SV detection from the PacBio sequencing platform was significantly more than that from ONT (*p* = 0.000173).

## Introduction

Structural variation (SV) is generally defined as a large-scale structural difference region of genomics DNA that are inherited and polymorphic in species (Mills et al., 2011). It accounts for the greatest number of divergent base pairs, including insertion (INS), deletion (DEL), inversion (INV), duplication (DUP), and translocation (TRA)/breakend (BND) (Weischenfeldt et al., 2013). SV represents a major form of genetic variations and contributes to polymorphic variations and phenotypes in organisms. Somatic SVs revealed that the deletion and rearrangement of chromosomal structure result in gene suppression and phenotypic transform, such as cancers (Stankiewicz and Lupski, 2010; Patel et al., 2014) and neurological disorders (Weischenfeldt et al., 2013; Brand et al., 2014). The genomic deletion of *D. melanogaster* had an effect on sensory perception environmental interaction and glutathione transferase activity (Zichner et al., 2013) and played a role in improvement of tropical inbred maize production (Yang et al., 2019). Meanwhile, SVs were associated with the position and/or function of cis-regulatory elements including promoters and enhancers (Spielmann et al., 2018). Recently, the advances in SV detection technologies have greatly facilitated the fine resolution of SVs (Zichner et al., 2013; Imprialou et al., 2017; Jeffares et al., 2017).

The accuracy of SV detection is the fundamental precondition of downstream biological investigation. At present, SV is mainly detected from short paired-end reads generated from the next-generation sequencing platform. However, the short reads usually lack sensitivity. For example, SMRT-SV, a short-read assembly-based approach, can only detect 10% SVs in CHM1 (Huddleston et al., 2017). In addition, the SVs detected from short-read by tools DELLY, LUMPY, and Pindel had an average of 0.75, 0.62, and 0.55 sensitivity in fission yeast, respectively (Jeffares et al., 2017). The 1000 Genomes Project Consortium identified more than 20,000 unreported SVs from short read with the false-positive rate as high as 89% (Teo et al., 2012; English et al., 2014). In contrast to short-read sequencing data, the long-read data from Pacific Biosciences (PacBio) or Oxford Nanopore (ONT) sequencing platform provide an advantage potential to increase the reliability and resolution of SV detection. Long- read can span the SV breakpoints with high-confidence alignments due to the length of long read (> 10 kb) (Rhoads and Au, 2015; Sedlazeck et al., 2018). Thus, SVs can be well detected by using long read from PacBio or ONT sequencing technologies (Chaisson et al., 2015; Chaisson et al., 2019; De Coster and Van Broeckhoven, 2019). Furthermore, the long read can be used to identify complex SVs (Sedlazeck et al., 2018) that would be missed in using short-read data.

Recently, several SV detection tools were developed for long-read sequencing datasets, including Picky (Gong et al., 2018), Sniffles (Sedlazeck et al., 2018), PBHoney (English et al., 2014), smartie-sv (Kronenberg et al., 2018), and NanoSV (Cretu Stancu et al., 2017). The Picky software accurately identified inversion using nanopore long read (Gong et al., 2018). Sniffles successively scanned alignments to identify all types of SVs (Sedlazeck et al., 2018). PBHoney and smartie-sv detected INS and DEL from alignments (English et al., 2014; Kronenberg et al., 2018). NanoSV was used to identify structural genomic variations in alignments based on long-read sequencing data from ONT GridION, or PacBio RSII, or Sequel sequencing platforms (Cretu Stancu et al., 2017). These SV callers were all based on the alignments of long read. The diverse alignment tools may result in various detection types of SV. The effect of SV detection under different combinations of alignments and SV callers should be further evaluated.

**Figure 1:**
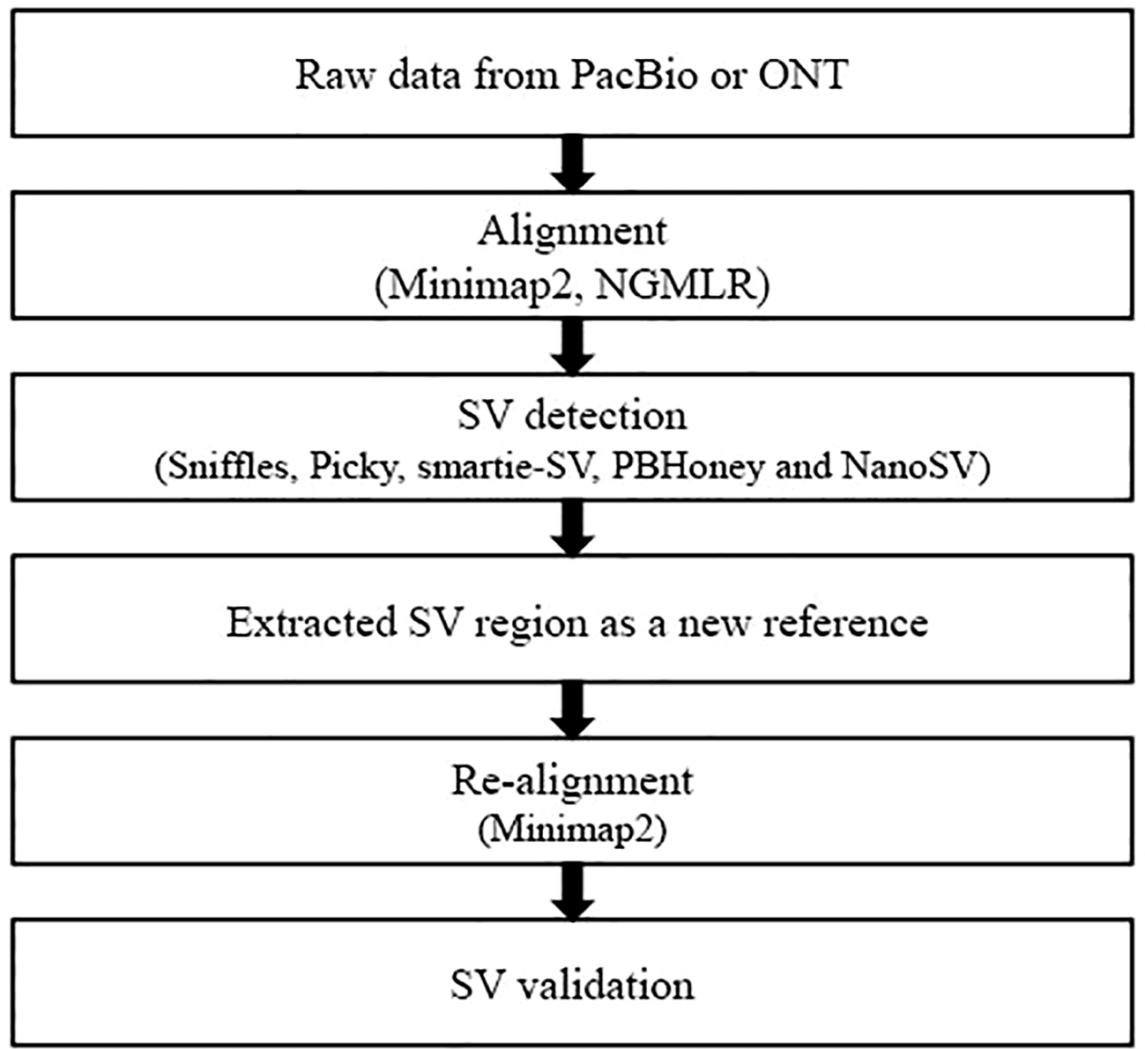
Overview of the evaluation pipeline in this study. Minimap2 (Li, 2018) and NGMLR (Sedlazeck et al., 2018) were used to perform alignment. Minimap2 aligned against reference genome with parameters ‘–MD -x map-pb/map-ont -R “@RG\tID:default\tSM : SAM” -a’ and NGMLR with the default parameters. SVs were identified by five callers, including Sniffles (Sedlazeck et al., 2018), Picky (Gong et al., 2018), smartie-sv (Kronenberg et al., 2018), PBHoney (English et al., 2014), and NanoSV (Cretu Stancu et al., 2017). Sniffles detected all types of SVs with parameters ‘–genotype –skip_parameter_estimation –min_support 10’ and employed a novel SV scoring scheme to exclude false SVs based on the size, position, type, and coverage of the candidate SVs (Sedlazeck et al., 2018). NanoSV was set ‘-s samtools’ to detect SVs and used the clustering of split reads to identify SV breakpoint junctions based on long-read sequencing data (Cretu Stancu et al., 2017). PBHoney considered both intra-read discordance and soft-clipped tails of long read (>10, 000 bp) to identify SVs (English et al., 2014). PBHoney, smartie-sv, and Picky were used to identify SVs with default parameters.

The main SV detection process includes two alignments and five callers. In this study, we systematically compared the combination SV detection tools of alignments and callers and developed an evaluation pipeline for SV detection from long-read datasets (**Figure 1**). We provided a comprehensive SV comparison analysis of five callers [Sniffles (Sedlazeck et al., 2018), Picky (Gong et al., 2018), smartie-sv (Kronenberg et al., 2018), PBHoney (English et al., 2014), and NanoSV (Cretu Stancu et al., 2017)] with two alignment tools [NGMLR (Sedlazeck et al., 2018) and minimap2 (Li, 2018)]. The long-read datasets from *Saccharomyces cerevisiae*, an ideal eukaryotic model organism (Ehrenreich and Magwene, 2017), were used to evaluate SV detection results under different combinations of alignment tools and callers, sample conditions, and three long-read sequencing instruments.

## Materials and Methods

### Data collection

Six long-read datasets from four yeast sample strains (Kagoshima no.2, BY4742, SY14 and NRRL Y-567) were collected from NCBI database (**Table S1**). The corresponding sample IDs were SAMD00082707, SAMN08364553, SAMN08364554, and SAMN09475318, respectively. SAMD00082707 was Kagoshima no. 2 strain from PRJDB5836 and used to brew the Japanese distilled spirit shochu. The long read of SAMD00082707 was sequenced 252-fold by three cells run on the PacBio RSII platform (Mori et al., 2017). The samples of SAMN08364553 and SAMN08364554 were wild-type strain BY4742 and SY14 from PRJNA429985 (Shao et al., 2018), which were sequenced by PacBio Sequel with 317-fold and 411-fold coverage, respectively. The sample SAMN09475318 from PRJNA477598 was the NRRL Y-567 strain, which was sequenced and generated three datasets in three instruments including PacBio Sequel, PacBio RSII, and ONT GridION (1D ligation kit and R9.5 flow cell) (Mcintyre et al., 2019). The collected six SRA format files were converted to FASTQ by using the NCBI SRA Toolkit (version 2.9.4).

### SV detection

Raw long-read alignment and SV calling were two key steps in SV detection. We used two alignment tools minimap2 (Li, 2018) and NGMLR (Sedlazeck et al., 2018) and five SV callers [Sniffles (Sedlazeck et al., 2018), Picky (Gong et al., 2018), smartie-sv (Kronenberg et al., 2018), PBHoney (English et al., 2014), and NanoSV (Cretu Stancu et al., 2017)] to perform the SV detection. To evaluate and compare the SV detection results from the combinations of alignment tools and SV callers, we established the following workflow (**Figure 1**): (1) FASTQ files of long read were used as input files to align against the *S. cerevisiae* S288C reference genome (R64) by minimap2 and NGMLR, respectively. (2) The alignment BAM files from step 1 were used to detect SVs by five callers: Sniffles, Picky, smartie-sv, PBHoney, and NanoSV. (3) Extract FASTA sequences of SV candidate regions as the reference sequences. (4) Re-align the raw reads to new reference and validate the candidate SVs. The theory of re-alignment to validate SVs is the double cross-verification method, which had been used in internal tandem duplication validation (Kluk et al., 2016) and novel miRNA gene identification from NGS datasets (Fehniger et al., 2010). The raw reads were re-aligned against the new reference (candidate SV regions) from the first alignment (step 3). The SV was positive if it was validated by re-alignment; otherwise, the SV was negative.

### Comparison SVs and bioinformatics analysis

To determine the common SVs from each of the two SV callers, we referred the previous criterion that defined the identical region as the intersect overlap of SV regions that accounted for at least 30% of each single SV region (Wong et al., 2010). BEDTools and Perl script were used to evaluate and compare the detected SVs. In order to compare SVs in five callers, we used the maximum SV interval that spanned the SV regions from all callers. The maximum SV interval referred to union of regions that spanned from the minimum of start positions to the maximum of end positions in five callers. The gene function under different sample conditions in yeast was annotated by DIVAD v6.8 (Huang Da et al., 2009). For comparison of detected SVs, we used the specificity and area under the curve (AUC) to measure SV callers. Specificity of each caller was calculated by true-negative SVs/real-negative SVs, and the common SVs detected by any two callers were used as real-positive sets. AUC was the area under a ROC curve that was created by plotting the true-positive rate (TPR) against the false-positive rate (FPR) at various threshold settings (Hanley and Mcneil, 1982). Then, Delong's test was used to compare two AUCs of combination alignments and callers (Delong et al., 1988). Besides, we used R 3.1.0 to perform the statistical analysis and figure drawing in this study.

## Results

### The performance of five callers in SV detection

SVs were detected by five callers for each dataset based on the alignment of raw long read, and a total of 1,077,819 SVs were detected from the raw detection, and 55.42% (597,359) of them were validated by re-alignment of raw long read in five callers (**Table 1**). Among them, 10,931, 3431, 129,412, 926,989, and 7056 SVs were detected by PBHoney, NanoSV, Picky, smartie-sv, and Sniffles, respectively (**Table 1**). We calculated the accuracy rate by validated SVs number/candidate SVs number. The accuracy of NanoSV, Sniffles, and smartie-sv was 68.67%, 60.47%, and 57.67%, respectively, which were more than the total average accuracy (55.42%). Smartie-SV detected the largest number of SVs (926,989), and PBHoney was with the highest average accuracy (89.04%). Although Picky detected 129,412 SVs and the number was only less than that of smartie-sv, the accuracy (35.85%) of Picky was the lowest. Furthermore, we considered the specificity and AUC of callers. The result showed that NanoSV (99.76%) and Sniffles (98.66%) had the top average specificities of five callers (**Table 1**). The average AUCs of PBHoney, NanoSV, Picky, smartie-sv, and Sniffles from six datasets were 62.10%, 52.68%, 55.33%, 70.33%, and 53.93%, respectively. Among them, smartie-sv was higher than the other four callers in most datasets (**Figures S1** and **S2**). Delong's test of each of the two callers showed that smartie-sv and PBHoney were significantly different from others, and NanoSV and Sniffles had non-significant difference in minimap2 (**Tables S2**–**S13**).

**Table 1:** The number of SVs detected by the combination of two alignment tools and five callers.

Sample_ID	Alignment tools	Five SVs callers																			
		PBHoney	Acc (%)	Spec (%)	AUC (%)	NanoSV	Acc (%)	Spec (%)	AUC (%)	Picky	Acc (%)	Spec (%)	AUC (%)	smartie-sv	Acc (%)	Spec (%)	AUC (%)	Sniffles	Acc (%)	Spec (%)	AUC (%)
SAMD00082707	minimap2	962/1440	66.81	97.7	64.5	291/492	59.15	99.69	52.13	9711/18,717	51.88	91.23	53.15	34,832/72,870	47.80	13.34	71.18	600/1010	59.41	98.04	51.4
	NGMLR	254/357	71.15	99.31	63.12	321/493	65.11	99.53	54.72	8306/14,591	56.93	94.37	53.16	25,943/47,460	54.66	8.4	74.95	376/546	68.86	98.38	53.95
SAMN08364553	minimap2	973/997	97.59	96.92	69.42	44/60	73.33	99.93	50.65	4027/8104	49.69	99.03	52.34	54,681/104,164	52.50	4.48	73.14	136/255	53.33	99.64	50.73
	NGMLR	2540/2680	94.78	93.8	67.88	30/43	69.77	99.95	50.07	3369/9561	35.24	97.68	52.35	53,516/81,092	65.99	8.86	70.72	184/248	74.19	99.71	50.43
SAMN08364554	minimap2	46/65	70.77	99.88	51.72	100/111	90.09	99.95	52.8	1898/4433	42.82	98.38	67.29	84,646/142,206	59.52	2.25	76.77	271/421	64.37	99.54	55.55
	NGMLR	974/1025	95.02	97.49	56.41	81/90	90.00	99.96	50.25	2978/7485	39.79	97.54	60	77,101/114,629	67.26	5.35	28.33	348/402	86.57	99.65	55.01
SAMN09475318_ont*	minimap2	399/467	85.44	96.25	62.79	185/299	61.87	99.73	53.93	1015/4625	21.95	98.33	51.55	10,074/19,509	51.64	8.58	72.74	283/487	58.11	97.11	54.83
	NGMLR	283/324	87.35	97.33	63.12	210/253	83.00	99.4	53.71	1540/2303	66.87	97.18	53.87	8297/14,717	56.38	9.07	75.63	256/410	62.44	97.02	54.94
SAMN09475318_rs2*	minimap2	395/460	85.87	98.39	61.01	267/435	61.38	99.7	54.38	4594/21,200	21.67	93.73	53.06	20,078/40,921	49.07	10.21	72.99	409/656	62.35	97.97	54.54
	NGMLR	267/308	86.69	98.94	63.53	281/396	70.96	99.48	54.17	4333/21,476	20.18	96.28	53.14	16,617/30,441	54.59	6.89	77.31	321/512	62.70	98.42	56.47
SAMN09475318_seq*	minimap2	463/497	93.16	98.85	59.16	243/366	66.39	99.94	54.16	1890/9254	20.42	98.83	57.93	79,413/14,8540	53.46	3.23	77.1	534/1201	44.46	99.15	55.99
	NGMLR	2177/2311	94.20	94.87	62.49	303/393	77.10	99.91	51.15	2738/7663	35.73	97.7	56.17	69,406/110,440	62.84	8.23	73.04	549/908	60.46	99.29	53.32

*ont, rs2, and seq presented the datasets from ONT GridION, PacBio RSII, and PacBio Sequel, respectively. Acc: the accuracy of validated SVs. Spec: the specificity of detecting SV by combination alignments and callers.

To further validate SVs from five callers, we calculated the common SVs for each dataset using five callers. In minimap2 alignment, a total of 46,396 SVs were detected by five callers in SAMD00082707 (**Table 1**). Among them, 956 common SVs were detected in any two callers, and PBHoney and smartie-sv had the highest amount of common SVs (450) (**Figure S3A**). There were 378 common SVs of a total of 59,861 SVs and 301 common SVs of a total of 86,961 in SAMN08364553 and SAMN08364554, respectively (**Table 1** and **Figures S3B, C**). In SAMN09475318, there were 11,956, 82,543, and 25,743 SVs detected in three long-read platforms (ONT GridION, PacBio Sequel, and PacBio RSII) (**Table 1** and **Figures S3D–F**), and PBHoney and smartie-sv also detected the highest amount of common SVs (161, 134, and 175). Meanwhile, we calculated common SVs based on NGMLR alignment (**Figure S4**). There were 35,200, 59,639, 81,482, 10,586, 21,819, and 75,173 SVs detected and validated in all six datasets (**Table 1**). PBHoney and smartie-sv also detected the most common SVs in SAMD00082707 (129), SAMN08364553 (872), SAMN09475318_ont (140), SAMN09475318_seq (606), and SAMN09475318_rs2 (149), respectively (**Figure S4**). Although the different SVs were detected in the five different callers with their own characteristics, the results from two callers, PBHoney and smartie-sv, that detected the most common SVs were consistent in minimap2 and NGMLR (**Figures S3** and **S4**).

### Comparison of detected SVs based on different alignments

We compared the SVs from minimap2 and NGMLR. The total raw detected SVs (604,262) and validated SVs (313,460) from minimap2 were more than the raw detected SVs (473,557) and validated SVs (283,899) in NGMLR, but the validated ratio of total SVs in NGMLR (59.95%) was higher than that in minimap2 (51.87%) (**Table 1**). Then, we integrated the overlap SVs of six datasets. In minimap2, the total 15,745 SVs were detected in five callers of all six datasets (547 in Sniffles, 1836 in PBHoney, 11,376 in smartie-sv, 219 in NanoSV, and 547 in Picky) (**Table 2** and **Figure S5A**). In NGMLR, we identified a total of 20,062 SVs in all six datasets (628 in Sniffles, 1824 in PBHoney, 15,455 in smartie-sv, 216 in NanoSV, and 1939 in Picky) (**Table 2** and **Figure S5B**). The number of detected SVs between minimap2 and NGMLR was tested by *t* test. The result showed that the difference between these two alignments was not significant (*p* = 0.9364), which suggested that the alignment tools did not have a significant effect on detecting SVs. However, the detected SV numbers in all five callers were obviously different (**Table 2**), and smartie-sv detected the most SVs in both alignments (**Tables 1** and **2**).

**Table 2:** The number of SVs from two alignments in five callers.

Callers	Minimap2	NGMLR	Common	Common/minimap2 (%)	Common/NGMLR (%)
Sniffles	547	628	244	44.61	38.83
PBHoney	1,836	1,824	749	40.80	41.06
smartie-sv	11,376	15,455	7,030	61.80	45.49
NanoSV	219	216	139	63.47	64.35
Picky	547	1,939	244	44.61	12.58

### Comparison of SVs in PacBio and Nanopore sequencing platforms

**Figure 2:**
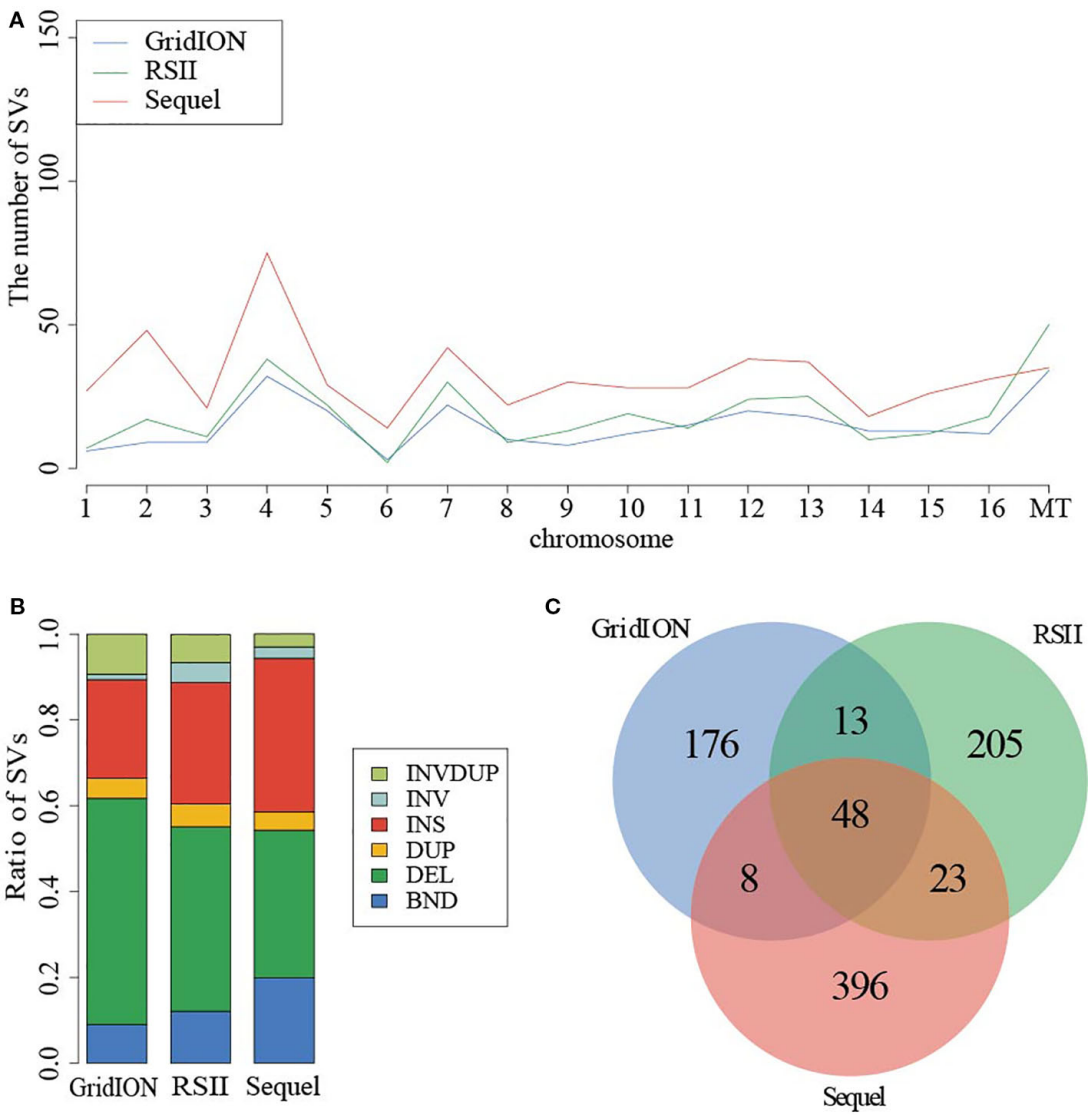
Comparison of SVs in PacBio and ONT sequencing platforms. **(A)** The distribution of SVs in chromosome. **(B)** The ratio of SV types from three instruments. **(C)** The common SVs among three instruments.

Given the detected SV types and accuracy of five callers, we used the NGMLR alignment tool and Sniffles caller to compare the sequencing platforms among PacBio RSII, PacBio Sequel, and ONT GridION in sample SAMN09475318. There were 321, 549, and 256 SVs detected in PacBio RSII, Sequel, and ONT GridION, respectively (**Figure 2**). The distribution of detected SVs among chromosomes were identical in these three long-read sequencing instruments, but the number of detected SVs from the PacBio sequencing platform was obviously higher than ONT (*p* = 0.000173) (**Figure 2A**). The DEL was the main SV type in the ONT platform (**Figure 2B** and **Table S14**). Furthermore, we detected 48 common SVs that related to 158 genes in three sequencing platforms (**Figure 2C**). We used DAVID to annotate these genes and found that the function of these genes was mainly in mitochondrial translational elongation and triplet codon–amino acid adaptor activity (**Table S15**).

### Comparison of SVs in different experimental conditions

**Figure 3:**
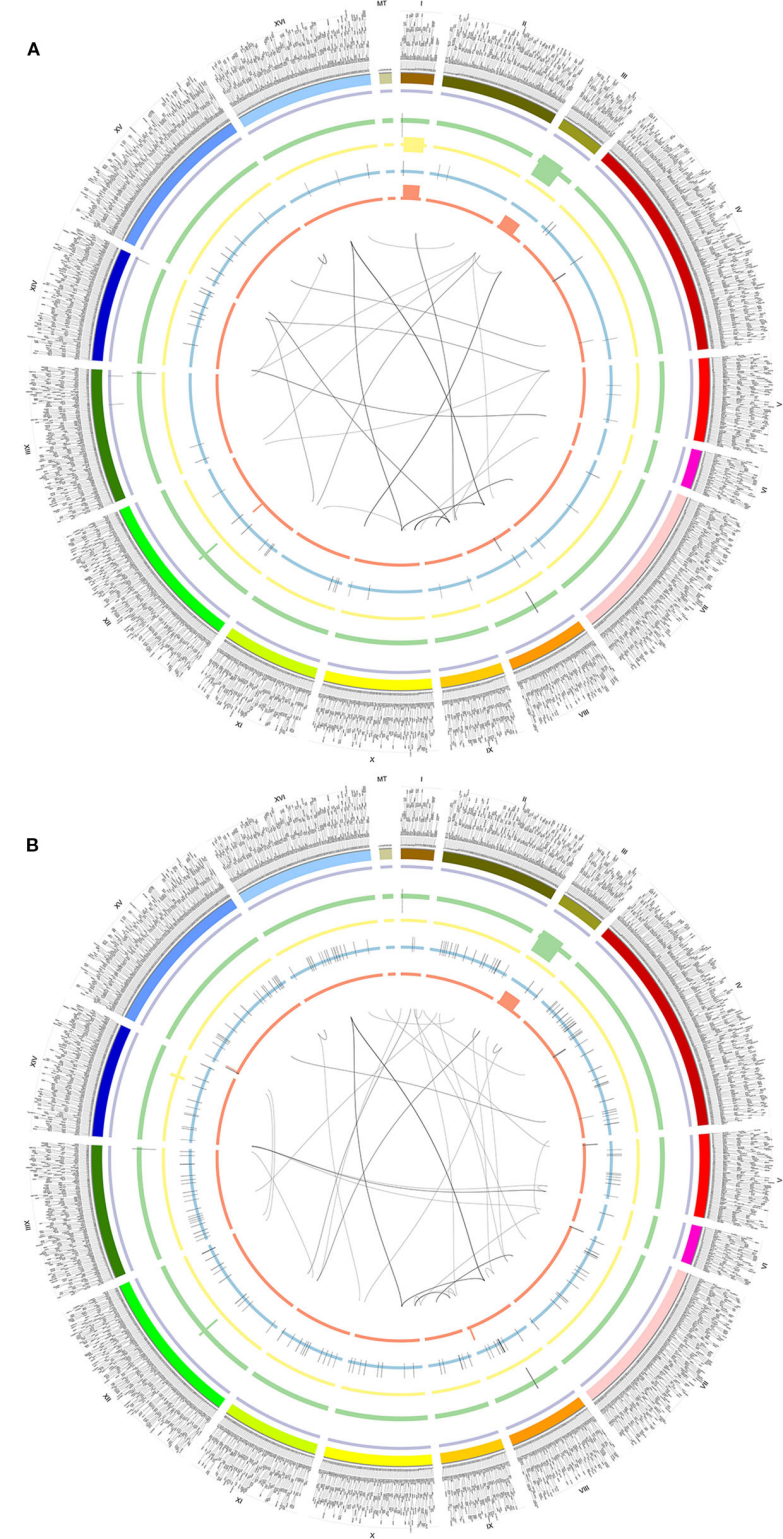
Circos of SVs detected by NGMLR and Sniffles in BY4742 **(A)** and SY14 **(B)**. From outer to inner, the first ring referred to chromosomes, and 2–6 were INVDUPs (purple), DUPs (green), INVs (yellow), INSs (blue), and DELs (orange). The inner lines referred to BNDs. The outer text referred to genes. **(A)** BY4742. **(B)** SY14.

To understand the function of SVs, we further dissected SVs from different yeast strains BY4742 (SAMN08364553) and SY14 (SAMN08364554) from the same study PRJNA429985 (**Table S1**). There were 184 and 348 SVs detected by the NGMLR alignment tool and Sniffles caller in BY4742 and SY14, respectively (**Table 1** and **Figure 3**). The subtypes of SVs including BND (59 and 59), DEL (13 and 18), DUP (21 and 30), INV (2 and 8), and INVDUP (40 and 44) were similar in BY4742 and SY14 (**Table S16**). However, INS (188) of strain SY14 was obviously more than that in BY4742 (49) (**Table S16**). This phenomenon was also confirmed in SV detection of minimap2 and Sniffles (**Figure S6A** and **Table S17**). The distribution of SVs in chromosomes between BY4742 and SY14 was close, and mitochondrial chromosome had the most SVs (**Figure S6B**). The number of SV subtypes in chromosome distribution between BY4742 and SY14 was tested by *t* test (**Table S18**). Among them, only INS was significant (*p* = 6.144E−05, **Table S18**), which was consistent with a previous result where INS of SY14 (188) was significantly more than that of BY4742 (49). Furthermore, we found 21 consistent SVs between BY4742 and SY14, and 14 of them related to 198 genes (**Table 3**). These genes were involved in mitochondrial translational elongation and triplet codon–amino acid adaptor activity by function annotation (**Table S19**). On the other hand, we found 160 and 322 special SVs in BY4742 and SY14, respectively (**Figure 3**). Based on the annotation of DAVID, special SVs of wild-type strain (BY4742) had an effect on the thiamine biosynthetic and metabolism process, and special SVs in SY14 strain were related to cytoskeleton and DNA recombination (**Table S20**). The SY14 strain was cultured by chromosome end-to-end fusions to create a single-chromosome yeast (Shao et al., 2018). Interestingly, we found that SVs on genes were related to DNA recombination, and more INSs of SVs were detected, which were rationality and reliability for the SY14 experimental condition.

**Table 3:** The common SVs in both BY4742 and SY14.

Chrom	Start	End	Type	Gene name
chr3	12542	200120	DUP	HMLALPHA2, HMLALPHA1, VAC17, MRC1, KRR1, FYV5, ADF1, MIC10, PRD1, PEX34, KAR4, RDT1, PBN1, LRE1, APA1, YCL049C, YCL048W-A, SPS22, POF1, EMC1, MGR1, YCL042W, GLK1, GID7, ATG22, SRO9, GFD2, GRX1, LSB5, MXR2, STE50, HIS4, BIK1, RNQ1, FUS1, HBN1, FRM2, AGP1, tE(UUC)C, YCL021W-A, YCL019W, YCL020W, SUP53, LEU2, NFS1, DCC1, YCL012C, GBP2, SGF29, ILV6, STP22, VMA9, snR43, LDB16, PGS1, YCL002C, RER1, YCL001W-A, YCL001W-B, YCR001W, CDC10, MRPL32, YCP4, CIT2, YCR006C, SUF2, YCR007C, tN(GUU)C, SAT4, ADY2, ADP1, PGK1, POL4, CTO1, snR33, SUF16, YCR016W, SRD1, tM(CAU)C, tK(CUU)C, MAK32, PET18, MAK31, HTL1, HSP30, YCR022C, YCR023C, SLM5, YCR024C-B, PMP1, NPP1, RHB1, tQ(UUG)C, FEN2, RIM1, SYP1, snR65, RPS14A, snR189, SNT1, ELO2, RRP43, RBK1, BUD5, MATALPHA2, CHA1, SPB1, PDI1, RRP7, KCC4, BUD3, CWH43, YCR025C, BPH1, PHO87, RVS161
chr3	12717	200620	DEL	HMLALPHA2, HMLALPHA1, VAC17, MRC1, KRR1, FYV5, ADF1, MIC10, PRD1, PEX34, KAR4, RDT1, PBN1, LRE1, APA1, YCL049C, YCL048W-A, SPS22, POF1, EMC1, MGR1, YCL042W, GLK1, GID7, ATG22, SRO9, GFD2, GRX1, LSB5, MXR2, STE50, HIS4, BIK1, RNQ1, FUS1, HBN1, FRM2, AGP1, tE(UUC)C, YCL021W-A, YCL019W, YCL020W, SUP53, LEU2, NFS1, DCC1, YCL012C, GBP2, SGF29, ILV6, STP22, VMA9, snR43, LDB16, PGS1, YCL002C, RER1, YCL001W-A, YCL001W-B, YCR001W, CDC10, MRPL32, YCP4, CIT2, YCR006C, SUF2, YCR007C, tN(GUU)C, SAT4, ADY2, ADP1, PGK1, POL4, CTO1, snR33, SUF16, YCR016W, SRD1, tM(CAU)C, tK(CUU)C, MAK32, PET18, MAK31, HTL1, HSP30, YCR022C, YCR023C, SLM5, YCR024C-B, PMP1, NPP1, RHB1, tQ(UUG)C, FEN2, RIM1, SYP1, snR65, RPS14A, snR189, SNT1, ELO2, RRP43, RBK1, BUD5, MATALPHA2, MATALPHA1, CHA1, SPB1, PDI1, RRP7, KCC4, BUD3, CWH43, YCR025C, BPH1, PHO87, RVS161
chr4	1335446	1335495	INS	PPZ2
chr4	528917	537428	DEL	ENA5, ENA2, ENA1
chr7	530002	530092	INS	MTL1
chr8	212266	216250	DUP	CUP1-1, YHR054C, RUF5-2, CUP1-2, RSC30, RUF5-1
chr9	25585	25655	INS	CSS1
chr11	64188	64283	INS	MNN4
chr12	451417	467360	DUP	RDN37-1, ETS2-1, RDN25-1, TAR1, ITS2-1, RDN58-1, ITS1-1, RDN18-1, ETS1-1, RDN5-1, RDN37-2, ETS2-2, RDN25-2, YLR154C-G, ITS2-2, RDN58-2, ITS1-2, RDN18-2, ETS1-2
chr13	908134	908705	DUP	YMR317W
chr14	415052	415283	INS	DBP2
chr16	528208	528340	INS	CIP1
MT	1	85779	DUP	tP(UGG)Q, 15S_RRNA, tW(UCA)Q, AI1, AI5_BETA, ATP8, ATP6, tE(UUC)Q, COB, BI4, BI3, BI2, OLI1, tS(UGA)Q2, 21S_RRNA, SCEI, tT(UGU)Q1, tC(GCA)Q, tH(GUG)Q, tL(UAA)Q, tQ(UUG)Q, tK(UUU)Q, tR(UCU)Q1, tG(UCC)Q, tD(GUC)Q, tS(GCU)Q1, tR(ACG)Q2, tA(UGC)Q, tI(GAU)Q, tY(GUA)Q, tN(GUU)Q, tM(CAU)Q1, COX2, Q0255, tF(GAA)Q, tT(UAG)Q2, tV(UAC)Q, COX3, tM(CAU)Q2, RPM1, COX1, AI5_ALPHA, AI4, AI3, AI2, VAR1
MT	41768	42012	INS	COB, BI4

## Discussion

Long-read sequencing datasets from the third-generation sequencing platform (PacBio or ONT) substantially increased the reliability and resolution of SV detection (Sedlazeck et al., 2018). In this study, we collected six long-read datasets of four samples in *S. cerevisiae* and detected SVs by using five callers including Sniffles, PBHoney, NanoSV, Picky, and smartie-sv from the alignment results of minimap2 and NGMLR. Our research firstly evaluated and compared the different SV callers for long-read datasets and developed an SV validation workflow by re-aligning the raw long read into SVs regions (**Figure 1**). All these popular, high-sensitivity SV detection callers were developed for long-read sequencing data. Picky exploited the long read of the ONT platform to identify the full spectrum of SVs with superior specificity and uncover micro-insertions based on a greedy seed-and-extension algorithm (Gong et al., 2018). Sniffles successively scanned alignments to identify unprecedentedly repeat-rich regions and complex nested events by putative variant scoring using several characteristics (Sedlazeck et al., 2018). PBHoney exploited the long read to detect larger structural variants using whole-genome PacBio RSII continuous long reads considering both intra-discordance and soft-clipped tails (English et al., 2014). Smartie-sv could query contigs against a reference genome and called structural variants including INS and DEL (Kronenberg et al., 2018). NanoSV was superior to short reads regarding detection of *de novo* chromothripsis rearrangements and enabled efficient phasing of genetic variations based on clustering of split reads. In this study, we compared and evaluated these tools.

The detection of SVs may be affected by different callers, alignment tools, sequencing instruments, and experimental conditions. We used minimap2 and NGMLR to align and compare the SV detection due to the running speed and special development purpose. Minimap2 was specifically developed to align long DNA or long mRNA sequences against a large reference for long read from the PacBio or ONT sequencing platform, which was one of the fastest long-read genomic or cDNA alignment tools at higher accuracy and surpassed most aligners. NGMLR was able to accurately align long single-molecule sequencing reads from PacBio and ONT with the goal of enabling precise SV detection, which was based on a convex scoring model to correct mapping linear alignments. Besides, the aligners BLASR, LAST, and BWA-SW were also used as alignment tools, but they did not fit the five callers well. There were no SVs in the combination BLASR and NanoSV or Sniffles, and LAST was not suitable for smartie-sv (**Table S21**).

In this study, five callers were compared under a specific aligned tool (minimap2 or NGMLR), and the alignment tools were compared by using the same caller. The uniform caller and alignment tool (NGMLR and Sniffles) were used to investigate the influence of different sequencing platforms in sample SAMN09475318 (PacBio RSII, Sequel, and ONT GridION) and to compare the experimental conditions between BY7472 (SAMN08364553) and SY14 (SAMN08364554). In short, the PBHoney was with the highest average accuracy (89.04%), and the accuracy of NanoSV, Sniffles, and smartie-sv was 68.67%, 60.47%, and 57.67%, respectively, which were higher than the average accuracy (55.42%). Smartie-SV detected the most SVs (926,989) and NanoSV was the least (3431). The alignment tools were without significant effect on detecting SVs. SVs from the PacBio sequencing platform was obviously higher than ONT (**Figure 3A**).

In the comparison of experimental conditions, we used the NGMLR as the alignment tool. Actually, minimap2 was also suitable for raw long read. The number of SVs detected by a combination of minimap2 and Sniffles was 136 and 271 SVs on strains BY4742 and SY14 (**Table 1**). The results from the combination of minimap2 and Sniffles also confirmed that INS of strain SY14 (124) was more than that in BY4742 (53) (**Table S17**). However, SVs detected by NanoSV in different conditions showed that the BND of strain SY14 (68) was more than that in BY4742 (13). We also found the difference from BY4742 and SY14 using smartie-sv with NGMLR and minimap2. The AUC (28.33%) of combination NGMLR and smartie-sv in SY14 was reduced. We further detailed the shared and special SVs and function of SV-related genes in BY4742 and SY14 under other combination alignments and callers. There were 11, 6, 55, 6583, 359, 10, 168, and 8148 shared SVs in combination minimap2 and NGMLR with PBHoney, NanoSV, smartie-sv, and Picky (**Table S22**). These gene-related shared SVs were enriched in function of ribosome biogenesis, cytoplasmic translation, retrotransposon nucleocapsid, cell cycle, mitochondrial translational elongation, and triplet codon–amino acid adaptor activity (**Table S23**). In BY4742, the genes of special SVs participated in mitochondrial translational elongation, triplet codon–amino acid adaptor activity, intron homing, hydrogen ion transmembrane transport, nucleic acid phosphodiester bond hydrolysis, and aminoacyl-tRNA biosynthesis, whereas genes of special SVs in SY14 played a role in oxidative phosphorylation and intracellular ribonucleoprotein complex (**Table S24**). This result further confirmed that the SV detection was greatly affected by the callers. The specificity and pertinence of callers need further study.

The alignment tool NGMLR was developed to align PacBio or Oxford Nanopore long read (standard and ultra-long) to a reference genome (Sedlazeck et al., 2018), which was suitable for five caller tools. The minimap2 was also an important alignment tool for long-read mapping. In this study, the difference between the alignments with minimap2 and NGMLR was not significant, and the type and position of SVs were mainly decided by caller tools. Although we had proposed the strategy to validate SVs by using re-alignment of raw long read for the different callers, the integration and improvement of the results from the different callers need further study.

## Data Availability Statement

Publicly available datasets were analyzed in this study. This data can be found here: PRJDB5836, PRJNA429985, PRJNA477598.

## Author Contributions

S-QX and X-MZ conceived the project and designed the experiments, M-WL and Z-BZ collected datasets and performed the bioinformatics analysis, and M-WL, S-QX, and YC wrote and revised the manuscript. All authors read and approved the final manuscript.

## Funding

This work was supported by grants from the National Natural Science Foundation of China (grant numbers 31760316, 31600667, and 31701146) and the Priming Scientific Research Foundation of Hainan University [grant number KYQD (ZR) 1721].

## Conflict of Interest

The authors declare that the research was conducted in the absence of any commercial or financial relationships that could be construed as a potential conflict of interest.
