# Supplementary material for: Evaluating Structural Variation Detection Tools for Long-Read Sequencing Datasets in Saccharomyces cerevisiae
Source: Front Genet. 2020 Mar 9;11:159. doi: 10.3389/fgene.2020.00159 (PMC7075250; doi:10.3389/fgene.2020.00159)
Supplement: Supplementary file 1 [file DataSheet_1.docx]

Supplementary materials:


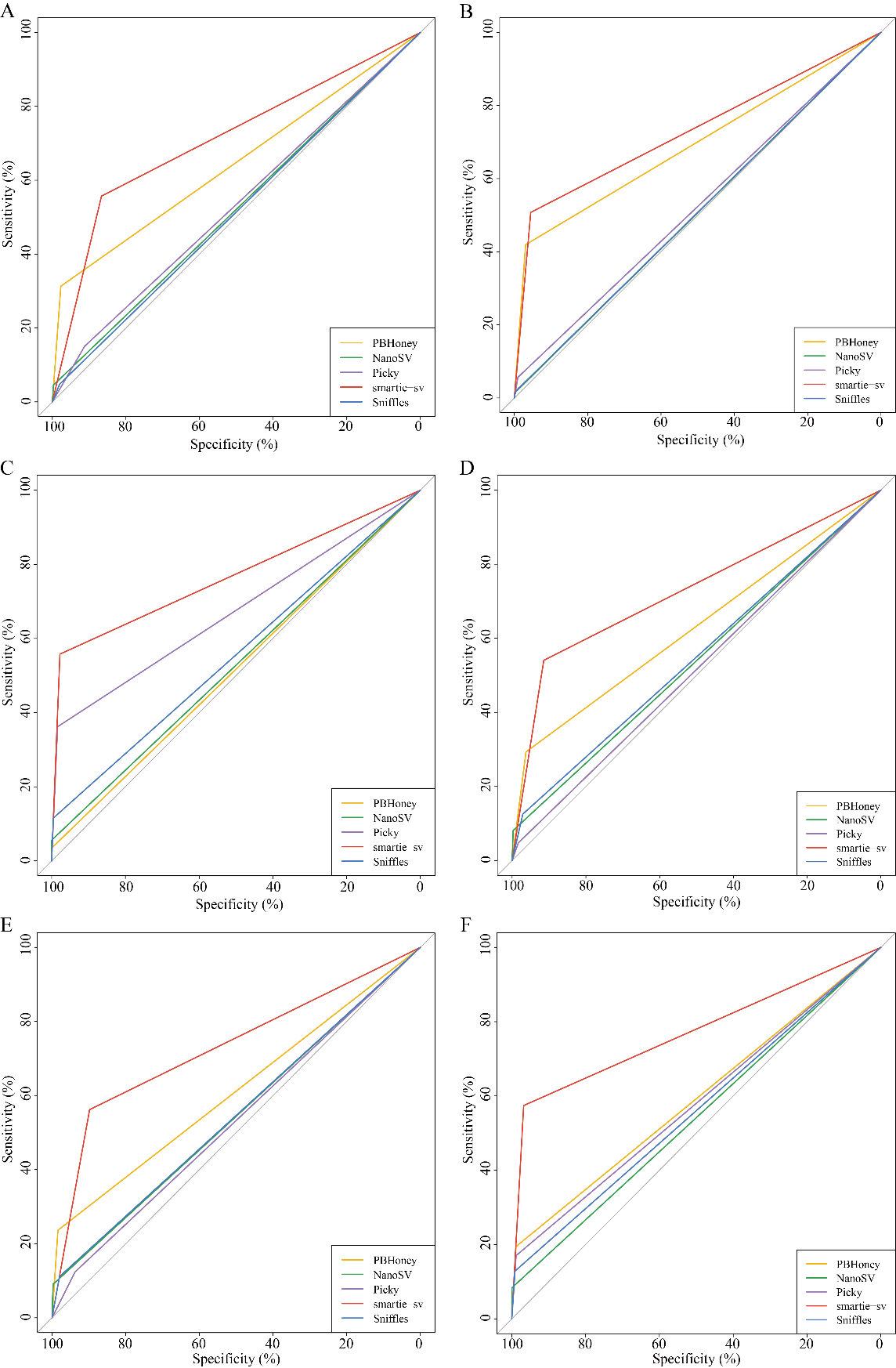


Figure S1. The ROC of SVs detected by five callers from minimap2 tools in each dataset. (A-C) SAMD00082707, SAMN08364553, SAMN08364554. (D-F) ONT GridION, PacBio Sequel and RSII sequencing of SAMN09475318.


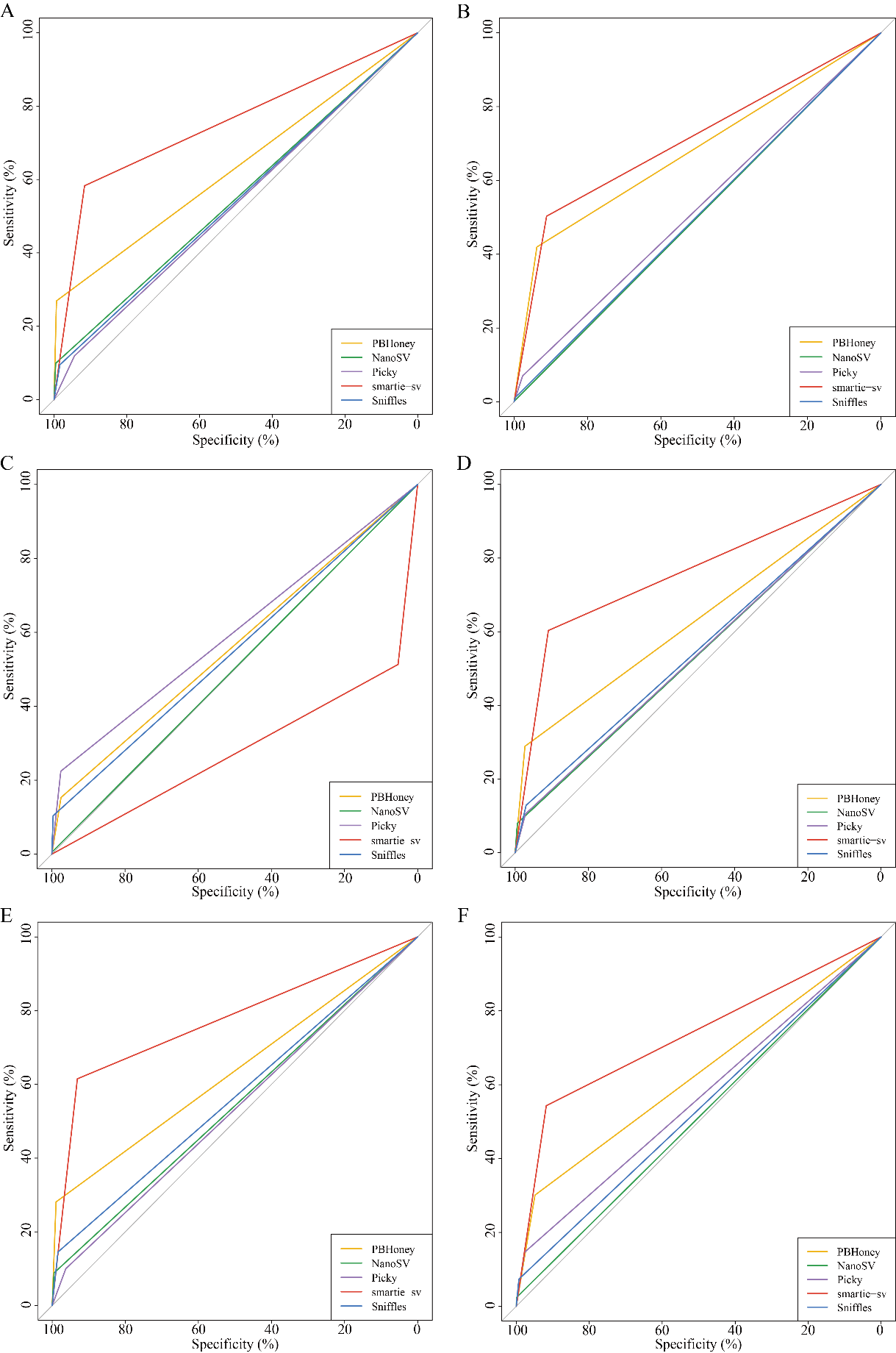


Figure S2. The ROC of SVs detected by five callers from NGMLR tools in each dataset. (A-C) SAMD00082707, SAMN08364553, SAMN08364554. (D-F) ONT GridION, PacBio Sequel and RSII sequencing of SAMN09475318.

**
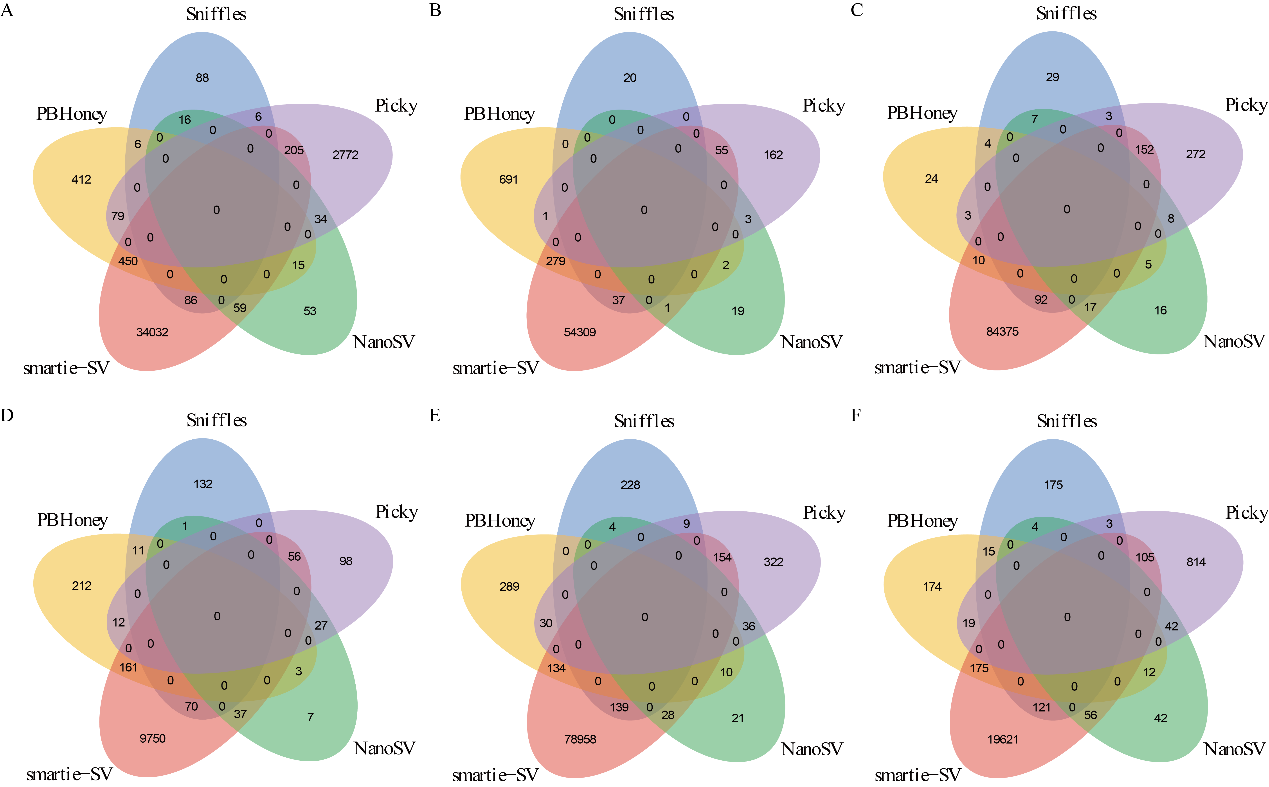
**

Figure S3. The venn diagram of SVs detected by five callers from minimap2 tools in each dataset. (A-C) SAMD00082707, SAMN08364553, SAMN08364554. (D-F) ONT GridION, PacBio Sequel and RSII sequencing of SAMN09475318.


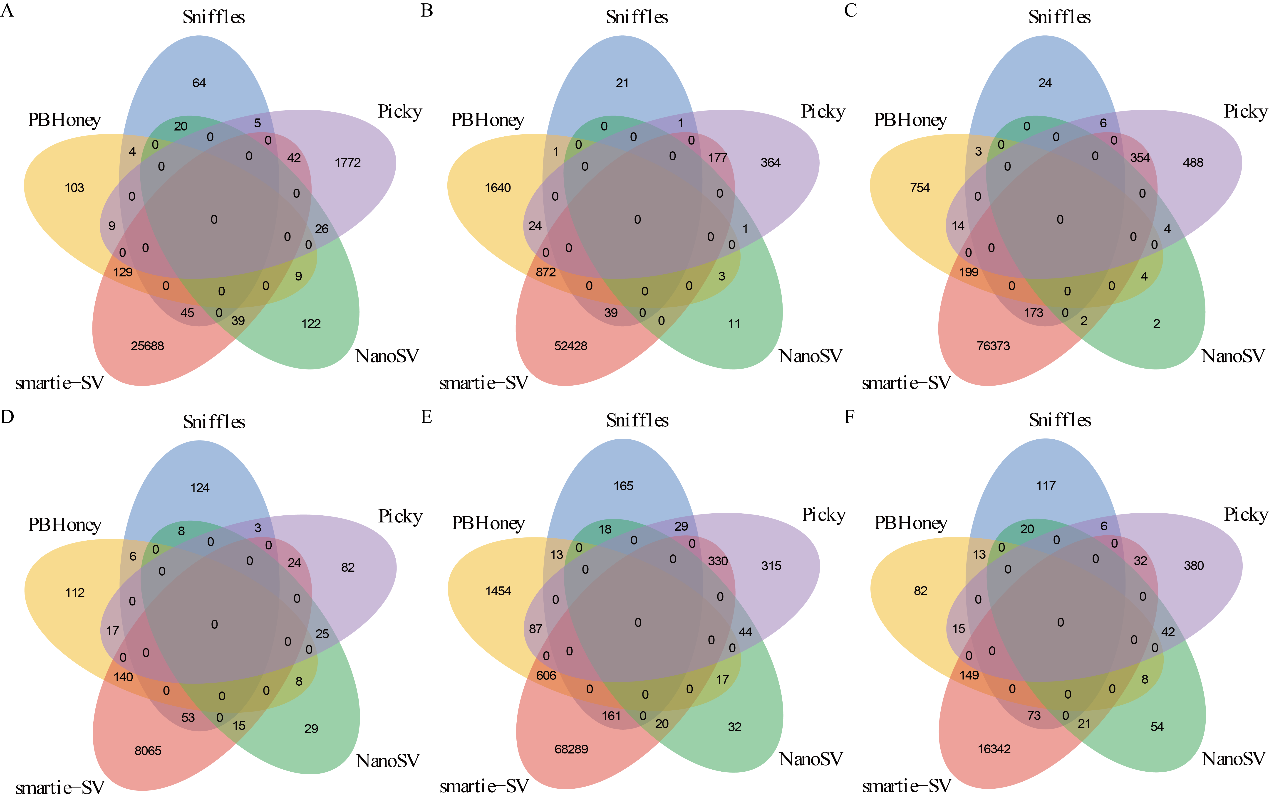


Figure S4. The venn diagram of SVs detected by five callers from NGMLR tools in each dataset. (A-C) SAMD00082707, SAMN08364553, SAMN08364554. (D-F) ONT GridION, PacBio Sequel and RSII sequencing of SAMN09475318.


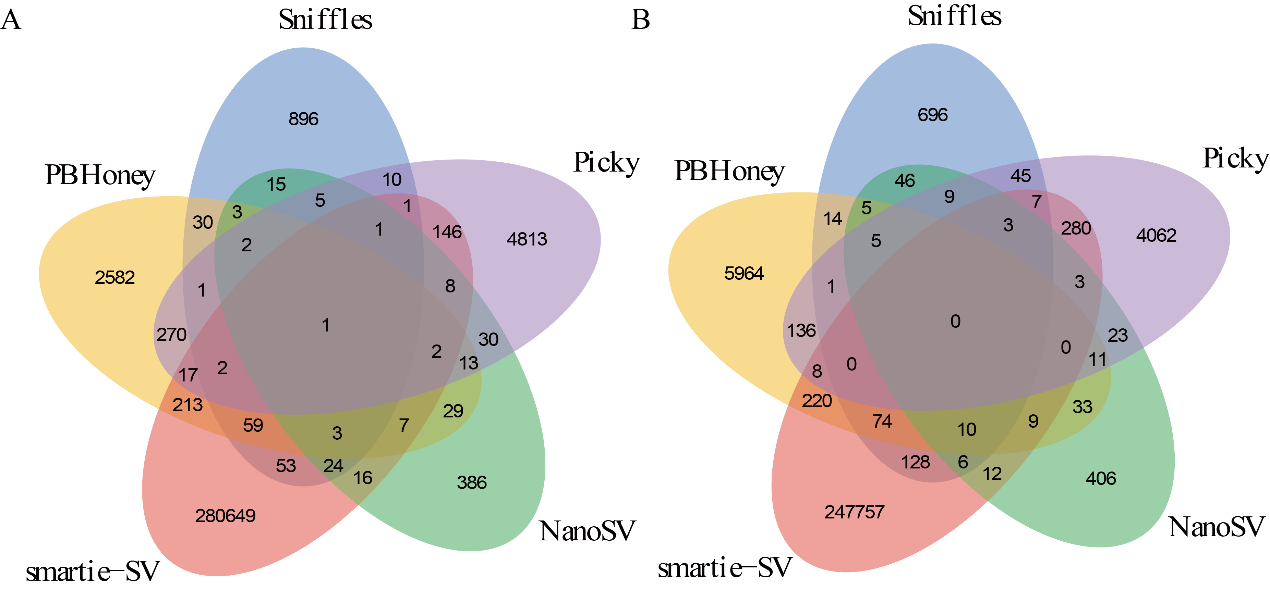


Figure S5. The venn diagram of SVs detected by five caller tools. (A) Mapping reference genome using minimap2. (B) Mapping reference genome using NGMLR.


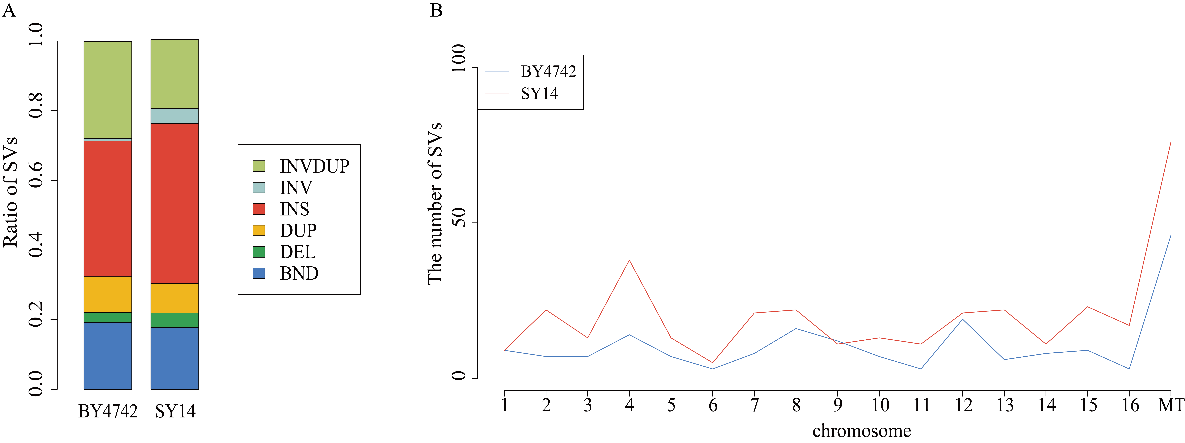


Figure S6. The distribution of SVs in different experimental conditions. (A) The types of SV detecting by minimap2 and Sniffles. (B) The SV distribution of chromosomes detecting by NGMLR and Sniffles.

Table S2. The p-value of Delong’s test comparing AUCs by five callers with minimap2 in SAMD00082707

| Callers | NanoSV | Picky | smartie-sv | Sniffles |
| --- | --- | --- | --- | --- |
| PBHoney | 2.20E-16 | 2.20E-16 | 2.20E-16 | 2.20E-16 |
| NanoSV | - | 0.07598 | 2.20E-16 | 0.07383 |
| Picky | - | - | 2.20E-16 | 0.002654 |
| smartie-sv | - | - | - | 2.20E-16 |

Table S3. The p-value of Delong’s test comparing AUCs by five callers with NGMLR in SAMD00082707

| Callers | NanoSV | Picky | smartie-sv | Sniffles |
| --- | --- | --- | --- | --- |
| PBHoney | 1.61E-10 | 3.04E-13 | 2.20E-16 | 1.95E-12 |
| NanoSV | - | 0.1378 | 2.20E-16 | 0.4357 |
| Picky | - | - | 2.20E-16 | 0.4492 |
| smartie-sv | - | - | - | 2.20E-16 |

Table S4. The p-value of Delong’s test comparing AUCs by five callers with minimap2 in SAMN08364553

| Callers | NanoSV | Picky | smartie-sv | Sniffles |
| --- | --- | --- | --- | --- |
| PBHoney | 2.20E-16 | 2.20E-16 | 2.20E-16 | 2.20E-16 |
| NanoSV | - | 0.000964 | 2.20E-16 | 0.8072 |
| Picky | - | - | 2.20E-16 | 0.002485 |
| smartie-sv | - | - | - | 2.20E-16 |

Table S5. The p-value of Delong’s test comparing AUCs by five callers with NGMLR in SAMN08364553

| Caller Tool | NanoSV | Picky | smartie-sv | Sniffles |
| --- | --- | --- | --- | --- |
| PBHoney | 2.20E-16 | 2.20E-16 | 2.20E-16 | 2.20E-16 |
| NanoSV | - | 1.74E-15 | 2.20E-16 | 0.00479 |
| Picky | - | - | 2.20E-16 | 4.32E-10 |
| smartie-sv | - | - | - | 2.20E-16 |

Table S6. The p-value of Delong’s test comparing AUCs by five callers with minimap2 in SAMN08364554

| Callers | NanoSV | Picky | smartie-sv | Sniffles |
| --- | --- | --- | --- | --- |
| PBHoney | 1.92E-01 | 2.20E-16 | 2.20E-16 | 2.22E-04 |
| NanoSV | - | 2.20E-16 | 2.20E-16 | 0.007879 |
| Picky | - | - | 2.20E-16 | 2.72E-11 |
| smartie-sv | - | - | - | 2.20E-16 |

Table S7. The p-value of Delong’s test comparing AUCs by five callers with NGMLR in SAMN08364554

| Callers | NanoSV | Picky | smartie-sv | Sniffles |
| --- | --- | --- | --- | --- |
| PBHoney | 2.20E-16 | 2.53E-05 | 2.20E-16 | 4.75E-02 |
| NanoSV | - | 2.20E-16 | 2.20E-16 | 2.20E-16 |
| Picky | - | - | 2.20E-16 | 1.75E-10 |
| smartie-sv | - | - | - | 2.20E-16 |

Table S8. The p-value of Delong’s test comparing AUCs by five callers with minimap2 in SAMN09475318_na

| Caller Tool | NanoSV | Picky | smartie-sv | Sniffles |
| --- | --- | --- | --- | --- |
| PBHoney | 2.85E-13 | 2.20E-16 | 2.20E-16 | 1.81E-09 |
| NanoSV | - | 0.00169 | 2.20E-16 | 0.3402 |
| Picky | - | - | 2.20E-16 | 0.000121 |
| smartie-sv | - | - | - | 2.20E-16 |

Table S9. The p-value of Delong’s test comparing AUCs by five callers with NGMLR in SAMN09475318_na

| Caller Tool | NanoSV | Picky | smartie-sv | Sniffles |
| --- | --- | --- | --- | --- |
| PBHoney | 9.15E-13 | 2.84E-11 | 2.20E-16 | 1.68E-08 |
| NanoSV | - | 0.8728 | 2.20E-16 | 0.2396 |
| Picky | - | - | 2.20E-16 | 0.3371 |
| smartie-sv | - | - | - | 2.20E-16 |

Table S10. The p-value of Delong’s test comparing AUCs by five callers with minimap2 in SAMN09475318_rs

| Caller Tool | NanoSV | Picky | smartie-sv | Sniffles |
| --- | --- | --- | --- | --- |
| PBHoney | 2.26E-11 | 6.77E-14 | 2.20E-16 | 3.83E-10 |
| NanoSV | - | 0.1119 | 2.20E-16 | 0.8466 |
| Picky | - | - | 2.20E-16 | 0.09082 |
| smartie-sv | - | - | - | 2.20E-16 |

Table S11. The p-value of Delong’s test comparing AUCs by five callers with NGMLR in SAMN09475318_rs

| Caller Tool | NanoSV | Picky | smartie-sv | Sniffles |
| --- | --- | --- | --- | --- |
| PBHoney | 9.36E-14 | 7.02E-16 | 2.20E-16 | 3.94E-07 |
| NanoSV | - | 0.2755 | 2.20E-16 | 0.028 |
| Picky | - | - | 2.20E-16 | 0.001974 |
| smartie-sv | - | - | - | 2.20E-16 |

Table S12. The p-value of Delong’s test comparing AUCs by five callers with minimap2 in SAMN09475318_na

| Caller Tool | NanoSV | Picky | smartie-sv | Sniffles |
| --- | --- | --- | --- | --- |
| PBHoney | 1.41E-07 | 2.69E-01 | 2.20E-16 | 2.34E-03 |
| NanoSV | - | 3.70E-05 | 2.20E-16 | 0.02749 |
| Picky | - | - | 2.20E-16 | 0.05379 |
| smartie-sv | - | - | - | 2.20E-16 |

Table S13. The p-value of Delong’s test comparing AUCs by five callers with NGMLR in SAMN09475318_na

| Caller Tool | NanoSV | Picky | smartie-sv | Sniffles |
| --- | --- | --- | --- | --- |
| PBHoney | 2.20E-16 | 2.20E-16 | 2.20E-16 | 2.20E-16 |
| NanoSV | - | 2.20E-16 | 2.20E-16 | 3.24E-11 |
| Picky | - | - | 2.20E-16 | 7.35E-09 |
| smartie-sv | - | - | - | 2.20E-16 |
